# Emergent Magnetic Order in Superconducting FeS Induced by Trace Cr Doping

**DOI:** 10.3390/ma18092108

**Published:** 2025-05-04

**Authors:** Yangzhou Wang, Qianshuo Wang, Yanhao Dong, Jin Wang, Shu Chen, Zihan Wang, Fei Chen, Guixin Cao, Wei Ren, Jie Li, Wen Wan

**Affiliations:** 1 Materials Genome Institute, Shanghai University, Shanghai 200444, China; yzwang@shu.edu.cn (Y.W.); 23724543wang@shu.edu.cn (Q.W.); dyh020220@163.com (Y.D.); jinwang@shu.edu.cn (J.W.); wangzihan@shu.edu.cn (Z.W.); renwei@shu.edu.cn (W.R.); 2 College of Physics and Electronic Information Engineering, Zhejiang Institute of Photoelectronics & Zhejiang Institute for Advanced Light Source, Zhejiang Normal University, Jinhua 321004, China; chf001@zjnu.edu.cn; 3 Terahertz Technology Innovation Research Institute, Shanghai Key Lab of Modern Optical System, University of Shanghai for Science and Technology, Shanghai 200093, China; 4 School of Materials Science and Engineering, Shanghai University, Shanghai 200444, China

**Keywords:** iron sulphide, superconductivity, magnetic order, orbital reconstruction, Cr doping, anisotropy reduction

## Abstract

Multiband and nodal-like superconductivity (SC) with s- + d-wave pairing symmetry have implied that tetragonal iron sulphide (FeS) is a distinctive testbed for exploring unexpected electronic correlations. In particular, the low-moment disordered static magnetism originating from the Fe moment leads to the possibility of the coexistence of magnetic orders (MOs) in the superconducting ground state via the tuning of electronic configurations. Here, guided by density functional theory (DFT) calculations, we found that slightly substitutionally doped chromium (Cr) atoms in tetragonal FeS single crystals can induce both considerable d-orbital reconstruction around the Fermi surface and a local magnetic moment of 2.4 *µ*_B_ at each doping site, which could highly modulate the SC ground states of the host. On this basis, a clear magnetic transition and reduced anisotropy of SC were experimentally observed. In particular, SC can survive with a doping content below 0.05. This coexistence of SC and MOs suggests strong spin correlations between Cr dopants and the host through exchange coupling. Further, an electronic temperature-related phase diagram of FeS with Cr doping contents from 0 to 0.07 is also provided. These results demonstrate that the continuous injection of local moments can be a controllable method to use to tune collective orders in unconventional iron-based superconductors.

## 1. Introduction

The exploration of many-body effects in electronic materials has fascinated scientists for decades. Particularly, as two mutually exclusive collective orders in nature, superconductivity (SC) and magnetism were discovered in 1970 to be able to coexist [1]. Significantly, they are two key ingredients needed to create non-Abelian quasiparticle excitations for topological quantum computation [2,3,4]. For ferromagnetic (FM) ordering, all spins tend to align in the same direction due to the presence of an effective exchange field, which breaks up Cooper pairs in a singlet state [5,6,7,8]. However, recent research has proven that singlet pairs can be transformed into triplet pairs through the exchange field in the interface of superconductor–ferromagnet heterostructures due to the proximity effect and thus enable the coexistence of SC and FM [5,9,10,11]. Additionally, in spin-triplet superconductors, magnetic fluctuation has been demonstrated to be essential for the origin of their SC [12,13]. Particularly, recent studies on Fe(Se, Te) superconductors have provided compelling evidence for the coexistence of FM orders, where they observed a hysteretic magnetization with Fe impurities, owing the fact that the supercurrent is suggested to mediate the interaction between these magnetic impurities [6,14,15].

Prospectively, as one of the superconducting Fe-chalcogenide family members, nodal-gaped tetragonal FeS [16,17] (whose SC transition temperature (*T*_c_) is around 4.5 K) is promising in terms of exhibiting FM ordering [18,19]. Compared to other Fe-based chalcogenide superconductors that have provided a rich and unmatched framework for assessing the interplay between unconventional SC and correlated electronic states [20,21,22,23], the electronic structures and magnetic properties of FeS show anomalous sensitivity to a small variation in the chalcogen coordinate, resulting from the complete reconstruction of its Fermi surface topology [24,25]. It has been reported that both vacancies and excessive Fe atoms can largely alter *T*_c_ [26,27,28]. Additionally, the *T*_c_ of FeS is first found to decrease under pressure, followed by the re-emergence of SC with the formation of a second superconducting dome on a phase diagram [29,30]. Beyond intrinsic defects or pressure that can induce rich and unusual properties in FeS, the isovalent substitution of S by other chalcogens is generally an effective strategy to study its unconventional SC competition or coexistence with differential electronic orders [31,32,33,34]. For instance, Se substitutions in FeS have revealed the rapid depression of SC induced by impurity scattering, leading to the coexistence of unexpected electronic nematicity [32,34]. Meanwhile, the aliovalent substitution of Fe by other magnetic elements can largely regulate the electronic correlations of FeS, whose Van Hove singularity mainly originated from Fe 3*d* orbitals [35]. For instance, the substitution of Fe by Co and Ni atoms can induce the emergence of both antiferromagnetism (AFM) and weak FM in FeS crystals [36,37].

Based on first-principle calculations, this work revealed a considerable *d*-orbital reconstruction around the Fermi surface of the FeS host when it is 5.6% Cr-doped in a substitutional way. Notably, a local magnetic moment of 2.4 *µ*_B_ at each doping site was also obtained in this model. With this guidance, Cr-doped tetragonal FeS single crystals were successfully grown. Substitutional doping was demonstrated using X-ray diffraction (XRD), scanning transmission electron microscopy (STEM), selected area electron diffractions (SAEDs) and X-ray photoelectron spectroscopy (XPS) analysis. The evolution of the collective orders of Fe_1−*x*_Cr*_x_*S (0 ≤ *x* ≤ 0.07) corresponding to the stoichiometric ratio *x* was particularly studied using transport measurements, which display a magnetic transition from SC ground states. Particularly, the observed coexistence of SC and MOs with a Cr doping content below 0.05 suggests an enhanced exchange coupling between local spins and conduction electrons, the so-called Ruderman–Kittel–Kasuya–Yosida (RKKY) interaction [38,39,40], due to the increased metallicity induced by the redistribution of *d* bands across the Fermi level (*E*_F_). Such spatially redistributed bands also lead to the largely reduced anisotropy of SC compared to the original FeS. In the end, a temperature-related electronic phase diagram of Fe_1−*x*_Cr*_x_*S with an increase in *x* from 0 to 0.07 is also provided based on our experimental observations.

## 2. Materials and Methods

### 2.1. Sample Preparation

Tetragonal Fe_1−*x*_Cr*_x_*S crystals were grown using a hydrothermal reaction. Fe powders (Aladdin, purity 99.9%, Shanghai, China), Cr powders (Aladdin, purity 99.5%, Shanghai, China) and S powders (SCR, purity 99.999%, Shanghai, China) were mixed according to a certain stoichiometric ratio. The mixtures were ground and pressed into pellets and then sealed in vacuumed quartz tubes. These tubes with samples inside were put into muffle furnaces and slowly heated up to 400 °C for 24 h. Then, the products of Fe_0.8(1−*x*)_Cr_0.8*x*_S polycrystals were mixed with potassium (SCR, 99%) before putting them into an alumina crucible and slowly heating them up to 1030 °C for another 24 h. The details of this process are elaborated as follows. First, fresh potassium lumps were mixed with Fe_0.8(1-*x*)_Cr_0.8*x*_S polycrystals in an inert atmosphere (Ar/glovebox) to prevent oxidation. Then, the mixtures were sealed in quartz tubes inside the glovebox using vacuum transfer valves to prevent any reactions with oxygen and water. Thereafter, the quartz tubes were vacuumed by a pump before being sealed with quartz stopcocks, and then they were ready for the next heating process. These alumina crucibles were all sealed in double-layer vacuumed quartz tubes during heating. Thereafter, these samples were cooled to 730 °C at a rate of 3 °C/hour to obtain K_0.4_Fe_0.8(1−*x*)_Cr_0.8*x*_S single crystals. A further hydrothermal reaction was applied to remove K atoms to obtain tetragonal Fe_1−*x*_Cr*_x_*S single crystals. For the hydrothermal reaction, each batch of products was obtained by heating the mixture of 0.24 g K_0.4_Fe_0.8(1−*x*)_Cr_0.8*x*_S, 0.12 g NaOH (Aladdin, 97%), 0.2284 g thiourea (Aladdin, 99%) and 10 mL ultrapure water at 120 °C in a Teflon-lined high-pressure reaction kettle (25 mL) for 72 h. Lastly, these samples were taken out before naturally cooling them down to room temperature (the whole growth process is also illustrated in Figure S1). These products were filtered, washed with ultrapure water and dried at room temperature to obtain silver-white crystals with sizes around 3 mm × 3 mm and a thickness of about 0.05 mm. These obtained samples could be better protected in the commercial high-vacuum system of Fermi Instruments (Oxide-MBE-350, Shanghai, China).

### 2.2. Measurements

Single-crystal and powder X-ray diffraction (XRD) patterns were measured using a Desktop X-ray Diffractometer from Bruker AXS Corp (D2 phaser, Billerica, USA) in the 2*θ* range from 10° to 65°. DC magnetization was measured using a Quantum Design (QD) Superconducting Quantum Interference Device (MPMS-3 SQUID Magnetometer, San Diego, USA ). Resistivity measurements were performed on the QD PPMS using a standard four-probe configuration. The sample used for resistivity measurements was cut into strips, and four gold wires were attached to the strips using silver glue. All electrical and magnetization measurements were taken using in-plane DC current. X-ray photoelectron spectroscopy (XPS, SPECS Surface Nano Analysis GmbH, Berlin, Germany) spectra were obtained from the Axis Ultra DLD system of Kratos with a monochromatic Al Kα X-ray source in an ultrahigh vacuum chamber with a base pressure of 5 × 10^−9^ Torr. The atomic structures and related SAED patterns were acquired on a special aberration-corrected transmission electron microscope with STEM modes (THEMIS ETEM G3, Waltham, MA, USA).

### 2.3. DFT Calculations

All the density functional theory (DFT) calculations were carried out with the Vienna ab initio simulation package (VASP) at the level of the spin-polarized generalized gradient approximation (GGA) with the functional developed by Perdew–Burke–Ernzerhof [41]. The framework of the projector augmented wave (PAW) method [42,43] was adopted for the interaction between valence electrons and ionic cores. To describe the dispersion forces well, a vdWs correction (DFT-D3) was included [44]. In DFT calculations, the energy cutoff for the plane wave basis expansion was set to 500 eV, and the criterion for total energy convergence was set at 10^−5^ eV. All atoms were fully relaxed using the conjugated gradient method for energy minimization until the force on each atom became smaller than 0.01 eV/Å.

## 3. Results and Discussion

### 3.1. The Proposed Model and Electronic Properties of Cr-Doped FeS

**Figure 1:**
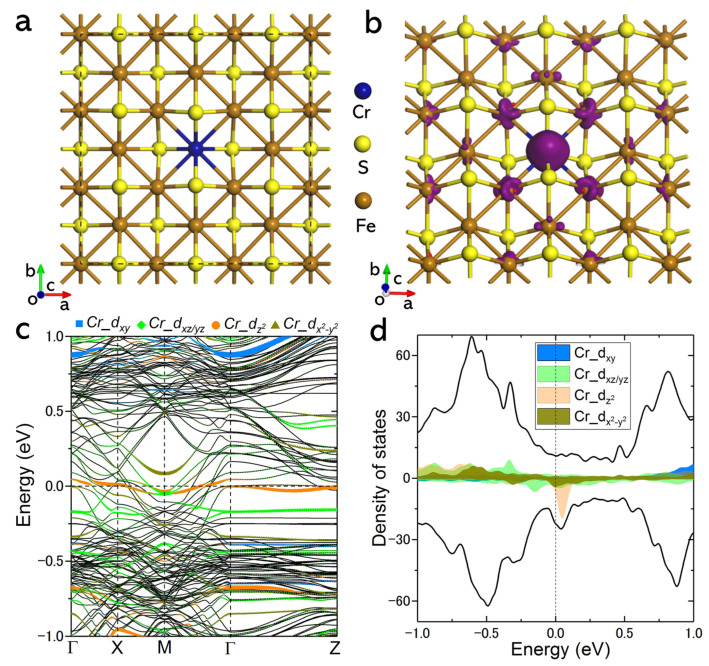
DFT-calculated electronic structures of Cr-doped tetragonal FeS. (**a**) Optimized 3 × 3 unit cell structure with single Cr dopant on Fe site. Only slight distortion can be observed in Cr-doped sublattice. (**b**) Band structures of this model. These band components contributed by Cr dopant are indicated by different colours. (**c**) Spin density around Cr dopants. Such doping-induced magnetic polarization can be extended to adjacent Fe atoms. (**d**) Projected spin-polarized density of states (DOS) originated from Cr. $d_{xz/\mathrm{yz}}$ and $d_{z^{2}}$ components that contributed mostly to local moment.

To understand the structure and electronic behaviour of tetragonal FeS with Cr dopants, we first designed models that have a 3 × 3 unit cell of FeS and a single Cr dopant (the stoichiometric ratio x is about 0.056). Generally, Cr atoms could be doped on different positions in FeS, including using the insertion method inside the van der Walls gap (approximate to absorption on the surface) and substitution on Fe sites. Among these models, the latter would induce a smaller out-of-plane lattice deformation than the absorbed case from the DFT calculations (see Table S1). The substituted case here is selected for a further study of its electronic behaviours (Figure 1). Figure 1a illustrates the optimized lattice of FeS with a single Cr dopant. Apart from a slight sublattice distortion around the doping site, the lattices of the host remain undisturbed, which largely enables structure stability after doping. The charges are found mostly distributed locally around the middle region between the Cr dopant and adjacent S atoms, which suggests rather strong interactions between them (see the charge difference density in Figure S2). The Bader charge shows that there are 1.02e electrons transferred from the Cr dopant to adjacent S atoms. Such charge spatial redistributions in covalently doped lattices commonly may give some signs of changes in orbital orientations, which could largely alter the band structures of the host, as indicated in Figure 1b. Compared to the calculated original FeS bands (see Figure S3) that show three typical bands across E_F_, Cr doping can yield even more new bands to increase the density state and thus enhance metallicity. Particularly, these new bands adjacent to E_F_ mostly originate from the d-orbitals of Cr (as seen from the coloured components $d_{xy}$, $d_{xz/\mathrm{yz}}$, $d_{z^{2}}$, $d_{x^{2}-y^{2}}$). Significantly, the $d_{xz/\mathrm{yz}}$ and $d_{z^{2}}$ components, which mainly contribute to a local moment of about 2.0 *µ*_B_, as indicated by the spin density in Figure 1c and the projected spin-polarized DOS in Figure 1d, may largely regulate the suggested $d_{x^{2}-y^{2}}$-wave superconductivity in FeS [35]. Based on these discoveries, we carried out experimental explorations to understand the evolution of ground states on gradually Cr-doped superconducting FeS.

### 3.2. Crystal Structures and Transport Measurement Analysis on Cr-Doped FeS

**Figure 2:**
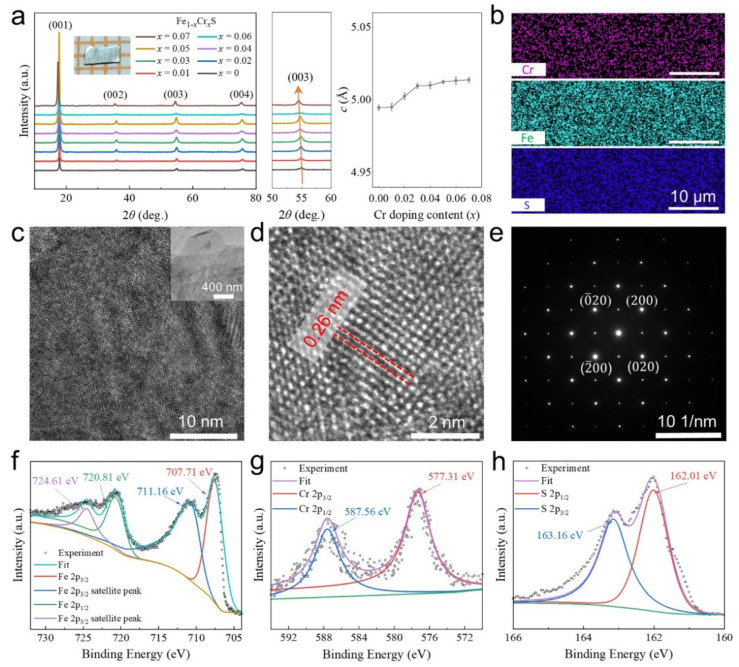
Characterization of tetragonal FeS with Cr substitutions. (**a**) XRD of Cr-doped FeS crystals and calculated *c*-axis parameters with *x* from 0 to 0.07. Highly preferred orientation of crystal structures is shown from these Bragg peaks of 00*l* (*l* = 1, 2, 3, 4) reflections, and slight left shift in these Bragg peaks is also observed, as seen on 003 reflections in centre panel as indicated by orange arrow, revealing some increase in *c*-axis parameters with addition of Cr dopants, as demonstrated in right panel. Inset shows one doped crystal with size of around 2.5 mm. (**b**) Element (Cr, Fe and S) distribution analysis on one doped sample with *x* = 0.05 using energy-dispersive X-ray spectroscopy (EDS). (**c**–**e**) STEM analysis on sample with same doping concentration as (**b**). Atomic stripes can be revealed in both large-scale (**c**) and high-resolution (**d**) STEM images; inset in (**c**) shows exfoliated film of this crystal. Value of 0.26 nm was obtained for sublattice parameter. SAED patterns (**e**) measured on same sample conform with features of pure FeS. (**f**–**h**) XPS analysis. Bonding states of Fe (**f**), Cr (**g**) and S (**h**) in our prepared crystals.

First, Cr-doped FeS crystals with a tetragonal phase are grown by using a hydrothermal reaction, similar to the growth of FeS single crystals [45]. The stoichiometry of these prepared crystals is established according to the mole fractions of precursors and further demonstrated by the energy-dispersive X-ray spectroscopy (EDS) analysis. Also, the structures of our prepared samples are studied by using XRD, STEM and XPS measurements (Figure 2). Our prepared crystals are first checked by SEM to exclude any potential existence of cracking that could affect the subsequent testing (Figure S4). Their highly preferred orientation of crystal structures is revealed by sharp Bragg peaks of 00*l* (*l* = 1, 2, 3, 4) reflections from XRD measurements (Figure 2a). These sharp 00*l* diffraction peaks, resulting from coherent scattering by 00*l* planes, evidence remarkable crystallographic orientation and structural periodicity, confirming the high single-crystalline quality. These Bragg peaks show similar features compared to those of the original FeS [46,47]. Doping-induced left shifts in these Bragg peaks are observed with increasing Cr contents, as indicated by the orange arrow along 003 peaks in the centre panel of Figure 2a. Such a slight increase in *c* parameters (right panel of Figure 2a) in our doped samples is much smaller than the van der Walls-intercalated cases [48,49], which exhibit the enlargement of *c* parameters on a scale of angstroms. Therefore, intercalation is not appropriate for Cr doping. Also, these prepared crystals can be easily cleaved into small pieces with regular shapes and flat surfaces (inset panel in Figure 2a), similar to the van der Waals-bonded FeS host. Apart from the Fe and S elements, doped Cr atoms are also homogeneously distributed in the crystal, as revealed by EDS mappings (Figure 2b).

The lattice structure of the host can be well maintained after relatively low substitutional doping (*x* ≤ 0.07), which is further demonstrated by STEM and SAED measurements. Due to their two-dimensional nature, Cr-doped FeS crystals can be easily cleaved into flakes with a few layers, as seen from the upper-right inset of Figure 2c, in which the sample has a Cr doping content of 0.05. Clear tetragonal atomic stripes can be identified in this large-scale high-resolution STEM image (Figure 2c), illustrating a considerable high quality of the FeS crystal after Cr doping. A sublattice parameter of 0.26 nm can be revealed from the enlarged atomic resolution image (Figure 2d). Such a value is consistent with that of the original FeS [45], providing evidence that Cr atoms are doped in a substitutional way, which largely preserves the tetragonal phase of the host with possible local changes in chemical environments. Additionally, clean SAED patterns (Figure 2e) measured within the regions of Figure 2c exhibit similar features as FeS without emerging extra patterns that are normally generated by some intercalation-induced superstructures [49]. The successful doping of Cr is further demonstrated by XPS analysis. Beyond the bonding states from the Fe (Figure 2f) and S (Figure 2h) elements, strong valence bands corresponding to the 2*p* orbital of Cr are also presented (Figure 2g). Due to a different bonding environment in the certain lattice, FeCr_2_S_4_ crystals with a hexagonal close-packed structure show obvious energy shifts compared to our Cr-doped FeS samples [50]. Here, we suggest that Cr atoms are doped into square-planar sublattices by substituting Fe atoms, forming the same tetragonal structures with a space group of P4/*nmm* [20,22]. Moreover, all these structural analyses comply with our calculated results on the energy-favoured Cr substitutionally doped model, which is also reasonably in line with other reports of metal-incorporated FeS [51].

### 3.3. Collective Ordering Transitions in Fe_1−x_Cr_x_S (0 ≤ x ≤ 0.07)

**Figure 3:**
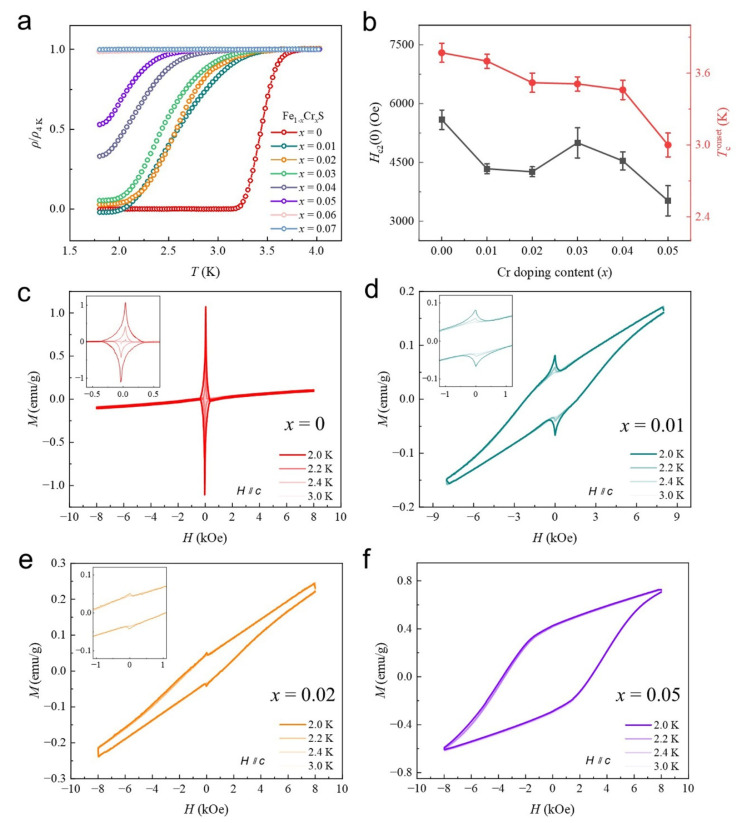
Transport measurements on Fe_1−*x*_Cr*_x_*S (0 ≤ *x* ≤ 0.07) crystals. (**a**) Temperature-dependent resistivity normalized to their resistivity at 4 K, revealing the gradual depression of *T*_c_ with increasing *x*. (**b**) The *x*-dependent evolution of *H*_c2_ and *T*_c_. Error bars are induced by fitting parameters. (**c**–**f**) Field-dependent magnetism (*M*-*H*) measurements on Fe_1−*x*_Cr*_x_*S crystals with *x* = 0 (**c**), *x* = 0.01 (**d**), *x* = 0.02 (**e**) and *x* = 0.05 (**f**) from 2.0 to 3.0 K. The opening of magnetic hysteresis is observed with an increase in *x*. Insets exhibit their enlarged curves.

According to our DFT calculations, Cr doping in FeS lattices yielded orbital reconstructions around *E*_F_. Such selective orbital reconstruction could induce unexpected changes in its ground states [52]. Thus, we analyzed the low-temperature (down to 1.8 K) transport characteristics of Fe_1−*x*_Cr*_x_*S with *x* varying from 0 to 0.07 (Figure 3). Our FeS host (*x* = 0) generally shows an SC transition around 4 K (see Figure S5), which will be the reference for studying their characteristic evolution with Cr doping. Their temperature-corresponded resistances (ρ/ρ_4K_) are displayed in Figure 3a, which shows a gradual decrease in the *T*_c_ transition with increased Cr doping, and SC faded away when *x* increased to 0.05. Besides *T*_c_, their upper critical fields (*H*_c2_) obtained by field-dependent *T*_c_ transition measurements (see Figure S6) also reveal a similar evolution. Considering that our DFT results provided evidence of a local magnetic moment generated around Cr dopants, we studied their field-dependent magnetism (*M*-*H*) behaviours on Fe_1−*x*_Cr*_x_*S crystals from 2.0 to 3.0 K. Compared to the original FeS samples that show a strong signal of diamagnetism (seen as two sharp peaks around 0 T, Figure 3c), hysteresis loops are accompanied below the SC transition temperatures for Cr-doped samples with *x* = 0.01 (Figure 3d) and 0.02 (Figure 3e). Such evidence of magnetic responses reveals that some types of MOs, typically FM [53,54], coexisted in SC states. When *x* reaches 0.05, diamagnetism degenerated into the magnetization hysteresis loop, conforming to the fading away of SC at this point. Compared to isovalent chalcogens (Se, Te) [31,32,33,34] and other magnetic elements (Co, Ni) [36,37] used for doping in FeS that either show slow or sudden changes on SC, our Cr-doped case may give rise to the possibility of the coexistence of SC with MOs within a controllable doping range. Additionally, for instance, in the sample of *x* = 0.01, the emerged hysteresis loops reclosed at room temperatures and appeared as paramagnetic, the same as the pure FeS (see Figure S7). Such *T*-related evolutions of these MOs are further demonstrated by *T*-dependent magnetism (*M*-*T*) measurements (see Figure S8), which reveals a clear FM transition from the initial paramagnetism states at temperatures around 67 K, corresponding with the *T*-related reclosing behaviours. The *x*-dependent evolution of coexistent electronic orders at 2 K provides clear evidence for the tuneability of collective orders via limited aliovalent substitutions.

### 3.4. Anisotropic Superconductivity

**Figure 4:**
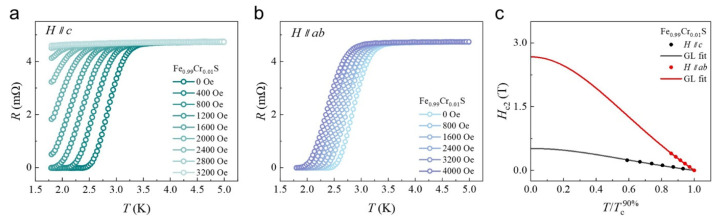
Upper critical field anisotropy in FeS crystal. (**a**,**b**) Field-dependent *T*_c_ degeneration of Fe_0.99_Cr_0.01_S perpendicular to and parallel with *ab* plane, respectively. (**c**) Upper critical fields of FeS in *ab* and *c* directions (fitted by GL formula).

Some possible mechanisms responsible for the currently observed coexistence of SC and MOs have been put forward to provide comprehensible scenarios for these systems. First, coexistence may arise when the Cooper pairs are in a triplet state, which is naturally favoured in the ferromagnetic state. Such magnetically mediated SC has been suggested in the pure metal ZrZn_2_, whose SC only occurs within the ferromagnetic phase [7]. Second, the coexistence of SC and FM manifesting in UGe_2_ is suggested to be mediated by itinerant *f* electrons, which can induce large magnetocrystalline anisotropy via strong spin–orbit interaction and is favourable for magnetic pairing in a triplet channel [55]. Thirdly, the recently reported heterointerface superconductor, which shows an in-plane magnetization hysteresis loop persisting above room temperature, is attributed to the oxygen vacancies that localize electrons in nearby heavy Ta 5*d* states through strong spin−orbit coupling effects [9]. Compared to these systems, both SC and the emerged MOs in our Cr-doped FeS could be predominantly derived from reconstructed bands at *E*_F_ [35,52], particularly the Fe and Cr 3*d* components. Thus, the doping-induced spatial redistribution of electronic states is expected to be coupled with pairing states, leading to a change in the anisotropy (Γ = $H_{\;c2}^{⫽ab}/H_{\;c2}^{⫽c}$) of SC compared to the original FeS (Γ ≈ 5.8) [46]. Therefore, the field-dependent *T*_c_ degenerations of Fe_0.99_Cr_0.01_S both perpendicular and parallel to its *ab* plane are investigated (Figure 4). The upper critical fields of FeS in the *ab* and *c* directions can be obtained by GL formula fit [56], which indicates 26,711 ($H_{\;c2}^{⫽ab}$) and 5155 Oe ($H_{\;c2}^{⫽c}$)) in these two directions; also, a value of 5.18 is obtained for anisotropy. The reduced anisotropy, resulting from the relatively weakened in-plane upper critical field of SC compared to the out-of-plane one, provides evidence that the *d*_xz/yz_ and *d*_z2_ bands contributed magnetic states from Cr dopants that could be coupled to the *d* bands of the host through the RKKY interaction and regulate the pairing states spatially [57], including the pairing coherence length (see Figure S9), which paves the way for the coexistence of MOs in the crystals.

### 3.5. Electron Correlations and Resulting Phase Diagram of Fe_1−x_Cr_x_S (0 ≤ x ≤ 0.07)

**Figure 5:**
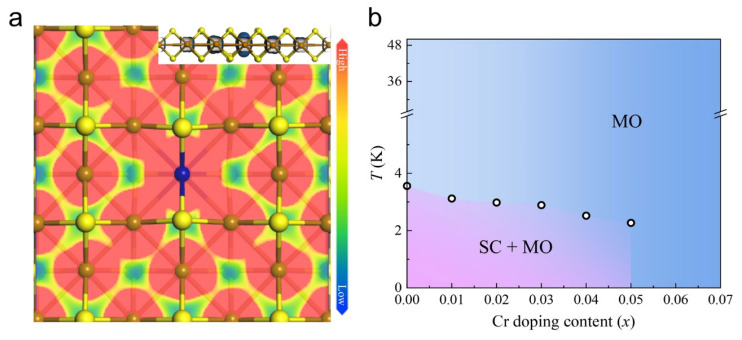
Cr doping-induced electron correlations and collective order transitions of Fe_1−*x*_Cr*_x_*S (0 ≤ *x* ≤ 0.07). (**a**) Spatial charge distribution of single Cr-doped FeS lattices within energy range of 0.1 eV above and below *E*_F_ (isosurface value is 1.0 × 10^−3^ e/Å^3^). (**b**) Phase diagram of Fe_1−*x*_Cr*_x_*S crystals with *x* ranging from 0 to 0.07. All dots indicate 90% *T*_c_.

To illustrate the *d* electron correlations and collective order transitions induced by Cr dopants, we evaluated the spatial charge distribution of single Cr-doped FeS lattices within the energy range of 0.1 eV above and below E_F_, where the *d*_xz/yz_ and *d*_z2_ bands are dominant (Figure 5). A relatively strong overlap of the charge distributions of the Cr atom and its adjacent Fe atoms is displayed (Figure 5a), which allows for possible channels for the RKKY interaction that results from the Kondo coupling between local spins and itinerant electrons, in spite of the fact that their electronic states are mostly localized (upper-right panel in Figure 5a). Based on this comprehension, a phase diagram of Fe_1−*x*_Cr*_x_*S corresponding to *T*_c_ and doping ratios (*x*) is also illustrated in Figure 5b for a direct full view of the evolution of collective orders within the range of their critical temperatures. Two phases including MOs and the coexistence of SC are compartmented. The narrow interval for the coexistence of SC and MOs reveals the effective long-range magnetic interactions between impurities in the finitely doped FeS superconductor.

Apart from our current study that provided the first full view of the tuning of the collective orders of tetragonal FeS with gradual Cr doping, some other issues regarding structural variation-related unconventional SC, as well as the subtle interaction between magnetism and the superconducting state in electron or hole-doped systems [58], still need to be further deliberated. Particularly, since the collective orders in our crystals are mainly related to the spatial distribution of 3*d* electrons from both Fe and Cr atoms, the scenario of how electron coupling or scattering induced the regulation of electronic states around Van Hove singularities [59] is still an open question for us to answer to understand the ordering mechanisms.

## 4. Conclusions

In summary, we systematically studied the structure and electronic properties of Cr-doped tetragonal FeS compounds (Fe_1−*x*_Cr*_x_*S). Firstly, guided by DFT calculations, we predicted that Cr dopants can induce both considerable *d*-orbital reconstruction and local magnetic moments, indicating a possible system for us to use to observe Kondo coupling between localized spins and itinerant electrons. Secondly, the structure and transport measurements demonstrated the substitutional doping of Cr atoms inside the FeS lattices and an MO transition from the SC state within a limited doping range from 0 to 0.07. Also, the doping-induced spatial redistribution of *d* bands was indicated by the reduced large anisotropy of *d*-wave SC. Thirdly, an RKKY-type interaction was suggested for the Kondo coupling between local spins and itinerant electrons in Fe_1−*x*_Cr*_x_*S, providing a comprehensive understanding of the electronic phase diagram of Fe_1−*x*_Cr*_x_*S (0 ≤ *x* ≤ 0.07). Our results offer a general methodology for the design of Fe-based compounds with tailored electronic textures that enable research on exotic forms of SC, including the coexistence of MOs, which offer potential application for the development of quantum electronic technologies [60]. Our results open the doors for further experimental investigations in Fe-based superconductors in the search of correlated normal states that include not only SC but also other electronic ordered states.

## Data Availability

The original contributions presented in this study are included in the article. Further inquiries can be directed to the corresponding authors.

## Supplementary Materials

The following supporting information can be downloaded at https://www.mdpi.com/article/10.3390/ma18092108/s1, Table S1: Lattice constants of bulk FeS, Cr-substituted FeS and Cr-absorbed FeS, respectively. Figure S1: Overview diagram of sample synthesis. Figure S2: The top (a) and (b) side views of the charge density difference. Figure S3: The original band structure of bulk FeS. Figure S4: A typical SEM image of the surface of as-grown Cr-doped FeS crystals. Figure S5: *T*-dependent resistivity and magnetism measurements on an FeS crystal. Figure S6: Field-dependent *T*_c_ transition measurements on Fe_1−*x*_Cr*_x_*S (0.01 ≤ *x* ≤ 0.07). Figure S7: *M*-*H* curves of FeS and Fe_0.99_Cr_0.01_S single crystals at 2 K and 300 K. Figure S8: *M*-*T* and *M*-*H* curves of Fe_0.99_Cr_0.01_S crystal. Figure S9: Doping-related Cooper pairing coherence length in Fe_1−*x*_Cr_*x*_S (0.01 ≤ *x* ≤ 0.07).

## Abbreviations

The following abbreviations are used in this manuscript:

SC	superconductivity
MOs	magnetic orders
DFT	density functional theory
FM	ferromagnetic
AFM	antiferromagnetism
XRD	X-ray diffraction
STEM	scanning transmission electron microscopy
SAEDs	selected area electron diffractions
XPS	X-ray photoelectron spectroscopy
RKKY	Ruderman–Kittel–Kasuya–Yosida
EF	Fermi level
DOS	density of states
EDS	energy-dispersive X-ray spectroscopy
QD	Quantum Design
SQUID	Superconducting Quantum Interference Device
VASP	Vienna ab initio simulation package
GGA	generalized gradient approximation
PAW	projector augmented wave
