# Supplementary material for: Emergent Magnetic Order in Superconducting FeS Induced by Trace Cr Doping
Source: Materials (Basel). 2025 May 4;18(9):2108. doi: 10.3390/ma18092108 (PMC12072792; doi:10.3390/ma18092108)
Supplement: Supplementary file 1 [file materials-18-02108-s001.zip › materials-3618899-supplementary.pdf]

materials

MDPI

Supplementary Materials

This file includes:

Table S1. Lattice constants of bulk FeS, Cr substituted FeS, and Cr absorbed FeS, respectively.

Figure S1. Overview diagram of sample synthesis.

Figure S2. The top (a) and (b) side views of charge density difference.

Figure S3. The original band structure of bulk FeS.

Figure S4. The typical SEM image of the surface of as-grown Cr-doped FeS crystals.

Figure S5. *T*-dependent resistivity and magnetism measurements on a FeS crystal.

Figure S6. Fields-dependent T_c transition measurements on $Fe_{1-x}Cr_xS$ ($0.01 \leq x \leq 0.07$).

Figure S7. *M*-*H* curves of FeS and $Fe_{0.99}Cr_{0.01}S$ single crystals at 2 K and 300 K.

Figure S8. *M*-*T* and *M*-*T* curves of the $Fe_{0.99}Cr_{0.01}S$ crystal.

Figure S9. Doping-related Cooper pairing coherence length in $Fe_{1-x}Cr_xS$ ($0.01 \leq x \leq 0.07$).

Table S1. The lattice constants of bulk FeS, Cr substituted FeS, and Cr absorbed FeS, respectively.

	Bulk FeS (Å)	Cr substituted FeS (Å)	Cr absorbed FeS (Å)
a = *b*	3.883	3.871	3.845
c	5.031	5.078	5.163

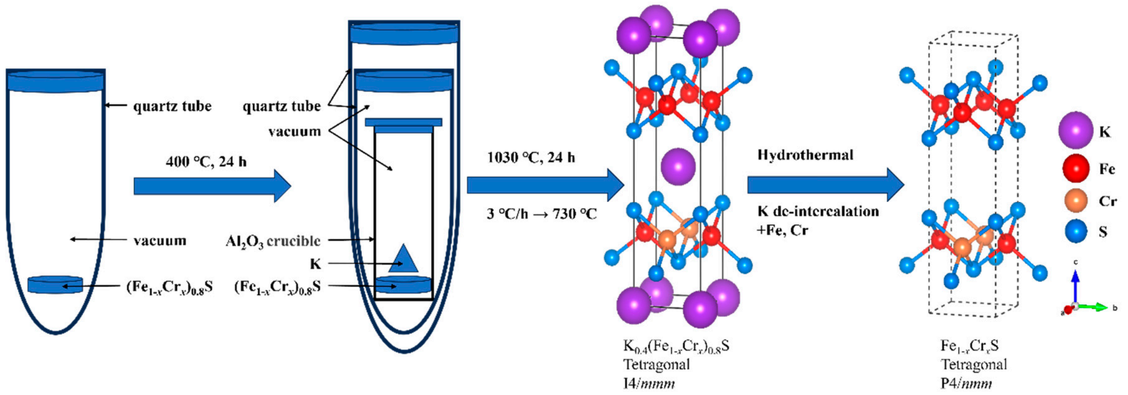

Figure S1. Overview diagram of sample synthesis.

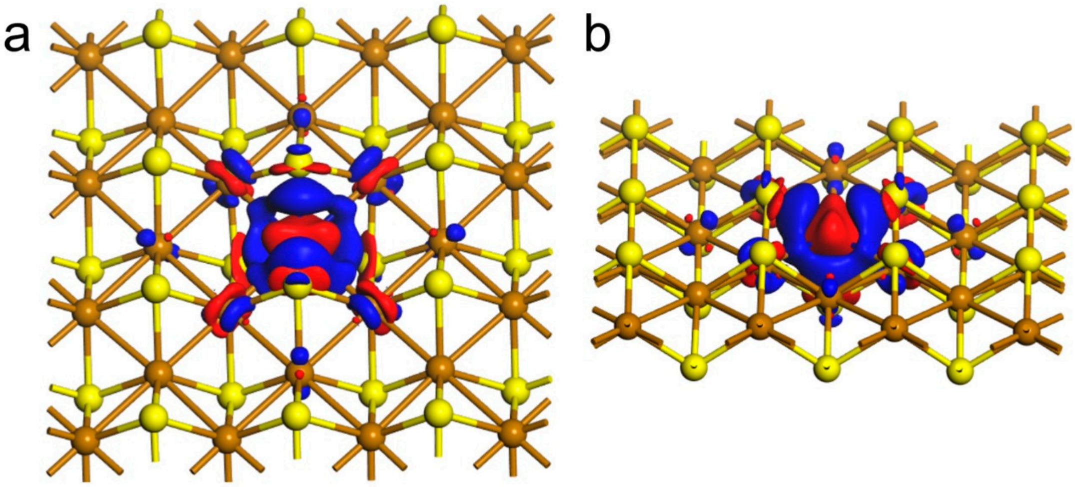

Figure S2. The top (a) and (b) side views of charge density difference. Blue and red colours represent charge accumulation and depletion (the isosurfaces are 1.0×10^{-3} e/Å^3), respectively.

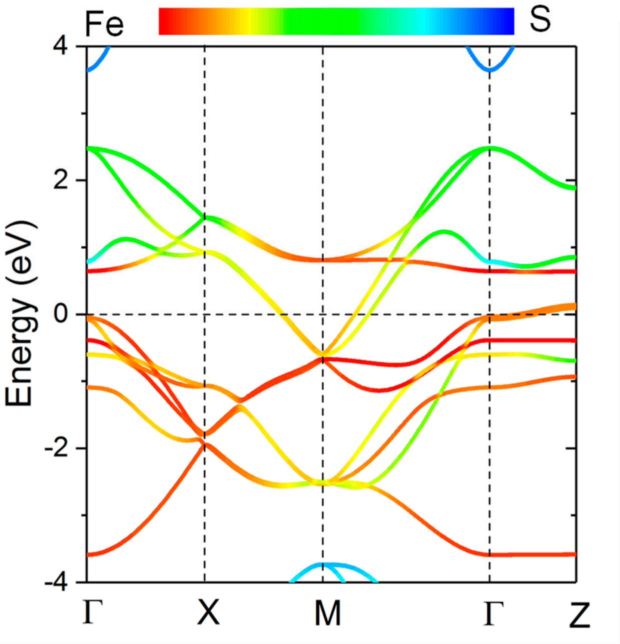

Figure S3. The original band structure of bulk FeS.

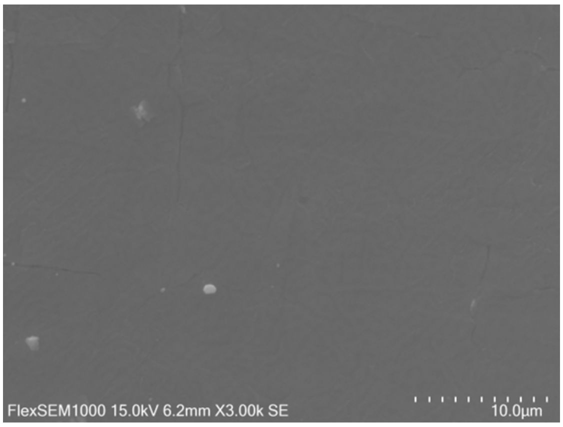

Figure S4. The typical SEM image of the surface of as-grown Cr-doped FeS crystals.

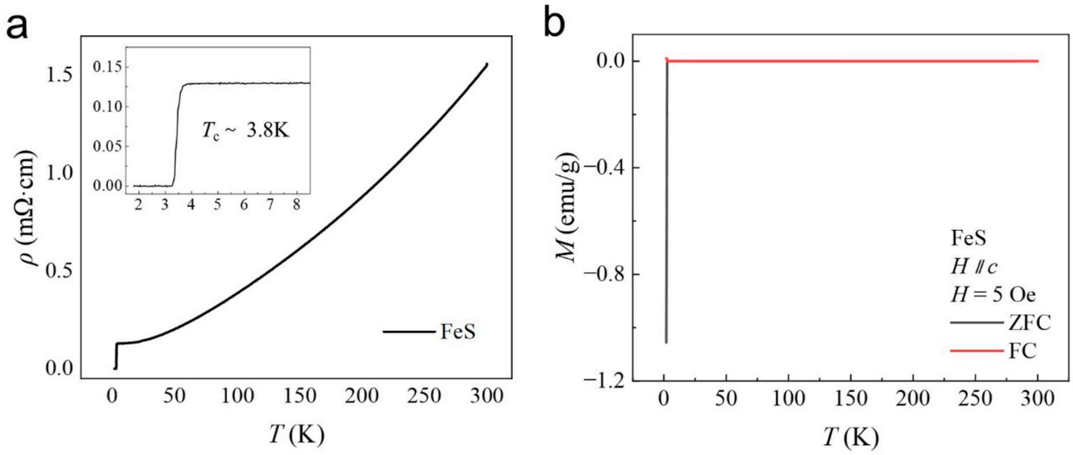

Figure S5. Temperature-dependent resistivity and magnetism measurements on our prepared FeS crystals. (a) Temperature-dependent resistivity from 300 K to 2 K, a sharp zero resistance drop is normally observed around 3.8 K. (b) A typical diamagnetic transition has also been shown from its magnetism measurement.

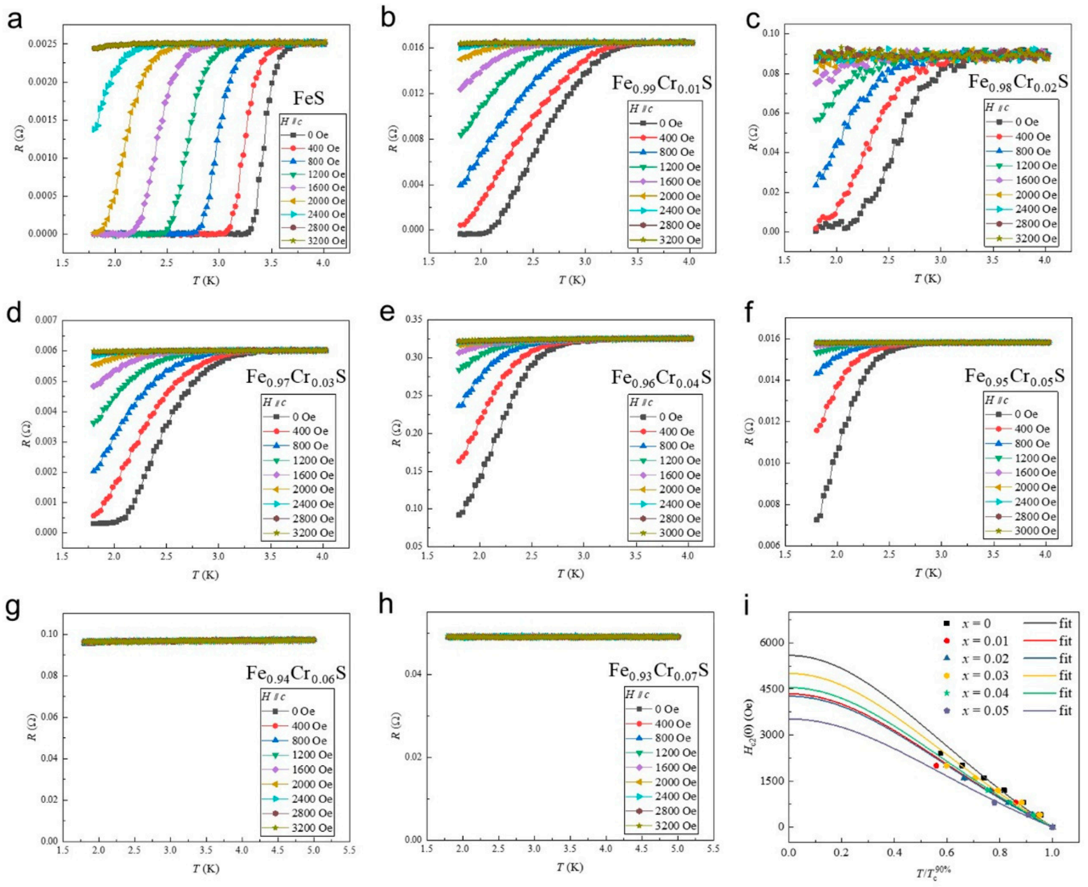

Figure S6. Fields-dependent T_c transition measurements on $Fe_{1-x}Cr_xS$ with x increasing from 0.01 to 0.07. The Fields-dependent T_c transition on (a) $x = 0$, (b) $x = 0.01$, (c) $x = 0.02$, (d) $x = 0.03$, (e) $x = 0.04$, (f) $x = 0.05$, (g) $x = 0.06$ and (h) $x = 0.07$. (i) The upper critical fields according to the Ginzburg-Landau formula [1,2]. The slight increase of both upper and lower critical fields for $x = 0.03$ indicates unusual features that Cooper-pairing mediated via spin fluctuations on bulk superconductivity coexisting with ferromagnetism [3].

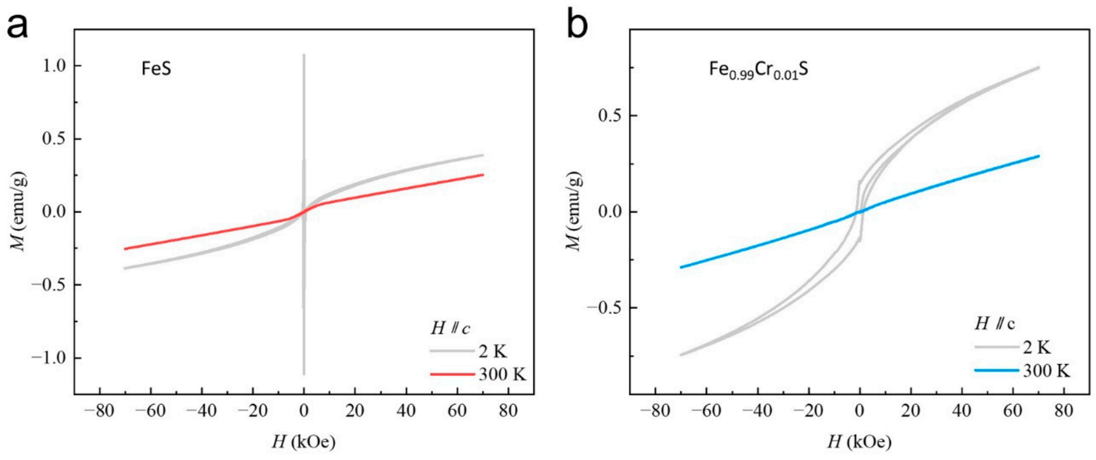

Figure S7. *M*-*H* curves of FeS and $Fe_{0.99}Cr_{0.01}S$ single crystals at 2 K and 300 K. (a) Diamagnetism and paramagnetism are shown on the original FeS crystal at 2 K and 300 K, respectively. A weak ferromagnetic background is also observed. (b) A clear hysteresis loop at 2 K and the paramagnetism are discovered on $Fe_{0.99}Cr_{0.01}S$.

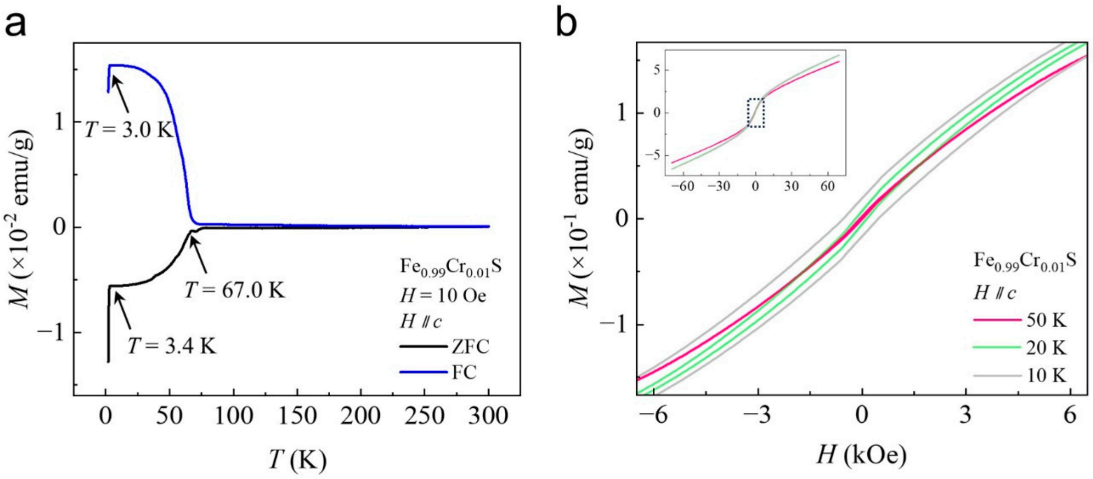

Figure S8. *M*-*T* and *M*-*H* curves of the $Fe_{0.99}Cr_{0.01}S$ crystal. (a) *M*-*T* curves (ZFC and FC) from 300 K to 2 K. Beyond the diamagnetism transition around 3.0 K due to SC, a clear FM transition from the initial paramagnetism is shown around 67 K. (b) *M*-*T* curves from 10 K to 50 K. A clear gradual reclosing of the hysteresis loop is shown.

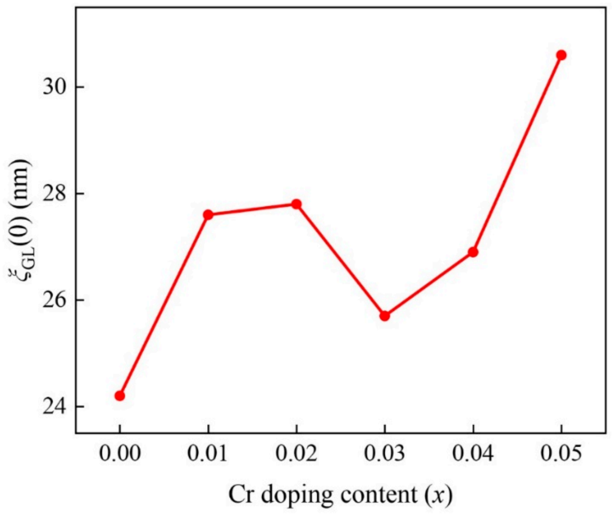

Figure S9. Doping-related Cooper pairing coherence length in $Fe_{1-x}Cr_xS$ obtained by the three-dimensional anisotropic Ginzburg-Landau (GL) theory $H_{c2}(0) = \frac{\Phi}{2\pi\xi^2_{GL}(T)}$ [4], where $\Phi_0 = 2.07\times10^{-7}$ Oe·cm².

References

1. Turkevich, L. A.; Klemm, R. A. Ginzburg-Landau theory of the upper critical field in filamentary superconductors. *Phys. Rev. B* **1979**, *19*, 2520–2539. https://doi.org/10.1103/PhysRevB.19.2520.
2. Askerzade, I. N. Ginzburg-Landau theory: the case of two-band superconductors. Phys.-Usp. **2006**, 49, 1003–1016. https://doi.org/10.1070/PU2006v049n10ABEH006055.
3. Thakur, G. S.; Fuchs, G.; Nenkov, K.; Haque, Z.; Gupta, L. C.; Ganguli, A. K. Coexistence of superconductivity and ferromagnetism in $Sr_{0.5}Ce_{0.5}FBiS_{2-x}Se_x$ ($x = 0.5$ and 1.0), a non-U material with $T_c < T_{FM}$. Sci Rep **2016**, 6, 37527. https://doi.org/10.1038/s41598-017-17637-y.
4. Tinkham, M; Emery, V. Introduction to superconductivity, Mac-Graw-Hill Inc., NewYork, NY, USA **1996**.
